# Neuromuscular Electrical Stimulation Under Deep Sedation Reduces the Incidence of ICU-Acquired Weakness in Critically Ill Patients With COVID-19 With Acute Respiratory Distress Syndrome

**DOI:** 10.7759/cureus.71029

**Published:** 2024-10-07

**Authors:** Saori Miyagishima, Masayuki Akatsuka, Hiroomi Tatsumi, Kanako Takahashi, Naofumi Bunya, Keigo Sawamoto, Eichi Narimatsu, Yoshiki Masuda

**Affiliations:** 1 Department of Rehabilitation, Division of Physical Therapy, Japan Healthcare University Faculty of Health Sciences, Sapporo, JPN; 2 Division of Rehabilitation, Sapporo Medical University Hospital, Sapporo, JPN; 3 Department of Intensive Care Medicine, Sapporo Medical University School of Medicine, Sapporo, JPN; 4 Department of Nephrology, Sapporo Hokushin Hospital, Sapporo, JPN; 5 Department of Emergency Medicine, Sapporo Medical University School of Medicine, Sapporo, JPN

**Keywords:** acute respiratory distress syndrome [ards], covid-19, critically ill patients, icu-acquired weakness, neuromuscular electrical stimulation

## Abstract

Background: The COVID-19 pandemic has led to an unprecedented increase in cases of acute respiratory distress syndrome (ARDS). In such cases, deep sedation using sedatives and muscle relaxants is commonly used to prevent patient self-inflicted lung injury during the early phase. However, such sedation limits the ability to perform early rehabilitation, leading to ICU-acquired muscle weakness (ICU-AW) and a worse prognosis.

Subjects: This study aimed to clarify the preventive effect of neuromuscular electrical stimulation (NMES) during deep sedation on ICU-AW and physical function at discharge in critically ill patients with COVID-19 with ARDS.

Methods: A retrospective, single-center study was conducted on patients admitted to the ICU or advanced critical care center with severe COVID-19 with ARDS between March 1, 2020, and March 31, 2022. Patients who were managed with the Richmond Agitation-Sedation Scale between −4 and −5 for at least three days were included. Patients in the NMES group received NMES within two days of deep sedation, whereas those in the non-NMES group did not. The primary endpoint was the incidence of ICU-AW at discharge from the ICU, and the secondary endpoints included physical activity levels, skeletal muscle mass index, time to active mobilization, and Barthel index (BI) at discharge. Statistical analyses included Pearson’s chi-squared test, Fisher’s exact test, and multiple logistic and linear regression analyses.

Results: Of the 129 patients, 68 (54 males and 14 females) were included after applying the exclusion criteria, with 38 in the NMES group and 30 in the non-NMES group. The incidence of ICU-AW was significantly lower in the NMES group (28.95% vs. 56.67%, p = 0.0211). NMES implementation (OR: 0.20, p = 0.03), ventilator weaning (OR: 0.10, p = 0.01), and duration of deep sedation (OR: 0.81, p < 0.01) were significant predictors of ICU-AW. Higher ICU Mobility Scale scores and shorter time to active mobilization were associated with a higher BI at discharge.

Conclusions: Early rehabilitation using NMES during deep sedation may prevent ICU-AW in critically ill patients with COVID-19 with ARDS. NMES is associated with a reduced risk of ICU-AW and improved functional independence at discharge. This procedure can be safely performed in sedated patients and may help prevent ICU-AW, supporting early mobilization strategies in ARDS rehabilitation.

## Introduction

The COVID-19 pandemic has led to an unprecedented surge in patients with acute respiratory distress syndrome (ARDS), significantly challenging our approach to critical care management strategies [1-4]. In managing ARDS, lung-protective strategies are crucial and often necessitate deep sedation and muscle relaxation to prevent patient self-inflicted lung injury. While this approach is vital for lung protection, it can inadvertently contribute to ICU-acquired weakness (ICU-AW), a condition associated with poor patient outcomes and difficult recovery.

Early mobilization is widely recognized as beneficial for critically ill patients, with the 2021 ARDS clinical practice guidelines in Japan emphasizing its importance [5]. However, the implementation of early mobilization becomes challenging during periods of deep sedation, creating uncertainty about appropriate interventions during this critical phase. Prolonged sedation and the use of muscle relaxants in critically ill patients have been strongly associated with the development of ICU-AW [6], which can significantly impair patient outcomes.

Recently, neuromuscular electrical stimulation (NMES) has emerged as a potential intervention to mitigate muscle weakness in critically ill patients. Several studies have reported its ability to contribute to the maintenance of muscle mass in ventilated patients. However, the preventive effect of NMES on ICU-AW, especially when used as early rehabilitation for deeply sedated patients with ARDS, remains unclear [7-9].

The rapid progression of muscle function decline in deeply sedated patients is notably challenging to reverse, highlighting the importance of prevention strategies. When patients are unable to achieve muscle contraction, they are more likely to develop disuse syndrome, which leads to a decline in their ability to perform basic activities of daily living. Therefore, the ideal strategy for the rehabilitation of patients with ARDS is to prevent the onset of ICU-AW in the early phase and to initiate exercise and weaning as soon as the patient’s general condition allows [10,11].

In light of these challenges, we have introduced NMES in addition to range-of-motion exercises and stretching as early mobilization during deep sedation. However, the efficacy of this approach in preventing ICU-AW and improving subsequent physical function and prognosis in deeply sedated patients with ARDS has not been fully elucidated.

Therefore, this study aimed to investigate the preventive effect of early rehabilitation using NMES on ICU-AW in deeply sedated patients with ARDS, with a specific focus on COVID-19 cases. Additionally, we aimed to identify factors influencing physical function at discharge in this patient population.

## Materials and methods

Study design and participants

This single-center, retrospective study was conducted at Sapporo Medical University Hospital (Sapporo, Japan) between March 1, 2020, and March 31, 2022. We included adult patients (≥18 years) diagnosed with severe COVID-19 with ARDS. Eligible patients were those who were managed with a Richmond Agitation-Sedation Scale score between −4 and −5 for at least three days in the intensive care unit (ICU). We excluded patients with significant pre-existing physical disabilities, those who died during hospitalization, and those who received rehabilitation intervention for fewer than seven days.

NMES intervention

Patients in the NMES group received NMES within two days of the initiation of deep sedation, in addition to standard care. We used a belt-electrode skeletal muscle electrical stimulation device (HOMER ION Laboratory Co., Ltd, Tokyo, Japan). NMES was applied to the entire lower limb, with the following parameters: pulse width 250 μs, frequency 1-20 Hz. The stimulation intensity was adjusted to achieve visible or palpable muscle contraction. NMES sessions were conducted daily for 20 minutes. The non-NMES group consisted of patients who did not have an attending physician’s permission before the start of NMES (for example, when stimuli such as delirium or agitation make it difficult to maintain calmness) or who needed more than three days to start NMES for some reason. Both the NMES and non-NMES groups received usual physical therapy interventions, including passive exercise, stretching, and positioning.

Data collection

We collected the following data from the electrical medical records of the patients: age, sex, body mass index at admission, severity of illness (APACHE II and SOFA scores), Charlson Comorbidity Index (CCI), ventilator-free days at 28 days, use of extracorporeal membrane oxygenation, supine positioning, use of muscle relaxants, and duration of deep sedation.

The primary outcome was the presence of ICU-AW at discharge from the ICU [12]. ICU-AW was diagnosed based on the Medical Research Council (MRC) total score (range 0-60) of <48 out of 60, assessed at least twice at intervals of 48 hours or more. Secondary outcomes included physical activity in the ICU, measured using the ICU Mobility Scale (IMS) (range 0-10) [13], skeletal muscle mass index (SMI), time to active mobilization, MRC score at ICU discharge, and Barthel index (BI) (range 0-100) at hospital discharge.

Statistical analysis

The primary and secondary outcomes were compared between the NMES group and the non-NMES group. We conducted a multiple logistic regression analysis to elucidate the effects of acute-phase rehabilitation using NMES on factors influencing the prevention of ICU-AW and physical function at discharge. For logistic regression, the presence of ICU-AW was used as the dependent variable, with APACHE II, NMES implementation (group), ventilator weaning, and deep sedation period as independent variables. For categorical variables, we used Fisher's Exact test to compare proportions between the NMES and non-NMES groups. For linear regression, the BI at discharge was used as the dependent variable, with ICU-AW, IMS, and time to active mobilization as independent variables. Before these analyses, we evaluated the potential correlations among independent variables and excluded highly correlated factors. We constructed a correlation matrix for all potential independent variables to check the strength and direction of their relationships. Pearson’s correlation coefficients were calculated for continuous variables, and Spearman’s rank correlation coefficients were used for ordinal variables. Among highly correlated variables with correlation coefficients |r| > 0.5, those deemed clinically or theoretically less important were excluded to minimize multicollinearity issues in the regression analysis. All statistical analyses were performed using R version 4.2.3 (R Foundation for Statistical Computing, Vienna, Austria), with a significance level set at 0.05.

Ethical considerations

This study was approved by the Clinical Research Review Committee of Sapporo Medical University Hospital (approval number: 332-187). This study was conducted as a retrospective analysis of existing clinical data. Due to the retrospective nature of the study, informed consent was waived, and an opt-out approach was implemented.

## Results

Basic characteristics

**Figure 1:**
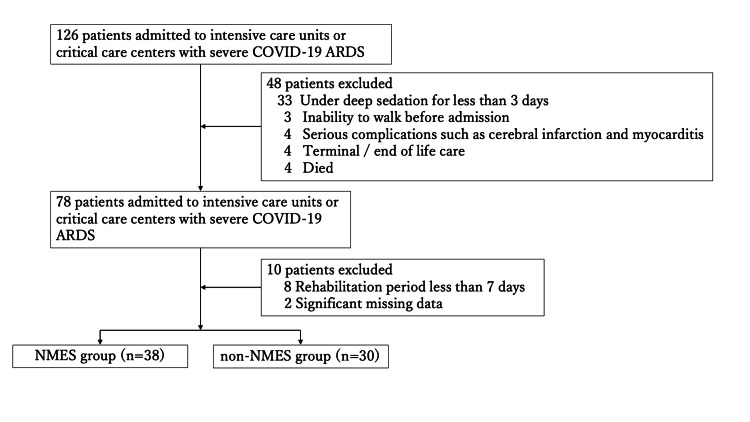
Flow chart of the patient selection process ARDS: acute respiratory distress syndrome, NMES: neuromuscular electrical stimulation

Of the 126 patients admitted to our ICU or advanced critical care center with COVID-19 with ARDS and who underwent acute-phase rehabilitation, 68 (54 males and 14 females) patients were included after applying the exclusion criteria. Of these, 38 patients were in the NMES group, and 30 were in the non-NMES group (Figure 1). Table 1 presents the patients' baseline characteristics, background factors, key treatment interventions, and clinical course. As shown, there were no statistically significant differences between the NMES group and the non-NMES group across these variables.

**Table 1:** Characteristics of the patients Data are presented as median [IQR]. APACHE, Acute Physiology and Chronic Health Evaluation; SOFA, Sequential Organ Failure Assessment; VV ECMO, Venovenous Extracorporeal Membrane Oxygenation

Variables	NMES group (n = 38)	non-NMES group (n = 30)	P value
Age (years)	57 [51—65]	59 [51—65]	0.915
Gender (male), n (%)	30 (78.9)	24 (80.0)	0.513
BMI at hospitalization (㎏/㎡)	28 [26—31]	30 [27—31]	0.496
APACHE II score	23 [19—26]	24 [18—27]	0.638
SOFA at ICU admission	8 [7—8]	8 [7—9]	0.178
Charlson Comorbidity Index	1 [0—1]	0 [0—1]	0.402
Prone positioning therapy, n(%)	38 (100)	30 (100)	0.332
VV-ECMO, n (%)	11 (28.9)	5 (16.7)	0.235
Neuromuscular blocking agents, n (%)	35 (92.1)	29 (96.7)	0.427
Ventilator weaning during hospitalization, n(%)	27 (71.1)	19 (63.3)	0.499
Ventilator-free days at 28 days	0 [0—16]	0 [0—20]	0.898
Duration of deep sedation	8 [4—17]	8 [4—24]	0.672

Primary and secondary outcomes

The presence of ICU-AW differed significantly between the groups (χ² = 5.318, df = 1, p = 0.02). Fisher’s exact test yielded similar results (p = 0.03; Table 2). The risk ratio analysis revealed that the incidence of ICU-AW was 28.95% (95%CI: 16.81%-45.09%) in the NMES group and 56.67% (95%CI: 38.84%-72.92%) in the non-NMES group. The relative risk was 0.51 (95%CI: 0.28-0.92), indicating that the risk of ICU-AW in the NMES group was approximately half that of the non-NMES group.

**Table 2:** Primary and secondary outcomes Data are presented as median [IQR]. ICU-AW, Intensive Care Unit-Acquired Weakness; IMS, Intensive Care Unit Mobility Scale; SMI, Skeletal Muscle mass Index; MRC, Medical Research Council score

Outcomes	NMES group (n = 38)	non-NMES group (n = 30)	P value
Primary Outcome			
ICU-AW, n (%)	11 (28.95)	17 (56.67)	0.021
Secondary outcomes			
IMS	6 [3—6]	3 [3—6]	0.048
SMI (㎏/㎡)	10.28 [9—12]	11.04 [10—12]	0.299
time to active mobilization	6 [4—15]	8 [7—21]	0.072
MRC score at ICU discharge	52 [41—60]	48 [35—52]	0.057
Barthel Index at discharge	53 [11—96]	23 [3—76]	0.153

The IMS was significantly higher in the NMES group compared to the non-NMES group (p = 0.048). However, there were no significant differences between the groups in terms of SMI, time to active mobilization, MRC score at ICU discharge, and BI at hospital discharge (Table 2).

Predictors of ICU-AW

Multiple logistic regression analysis identified significant predictors of the onset of ICU-AW (Table 3). NMES implementation (OR: 0.20, 95%CI: 0.05-0.89, p = 0.03), ventilator weaning (OR: 0.10, 95%CI: 0.02-0.50, p = 0.01), and the duration of deep sedation (OR: 0.81, 95%CI: 0.73-0.90, p < 0.01) were statistically significant predictors of ICU-AW. In contrast, the APACHE II score did not show statistical significance.

**Table 3:** Logistic regression results for predictors of ICU-AW APACHE, Acute Physiology and Chronic Health Evaluation; NMES, Neuromuscular Electrical Stimulation; ICU-AW, ICU-acquired muscle weakness

Variable	Std. Error	z value	Odds Ratio (95% CI)	Coefficient	p value
Intercept	2.631	0.204	1.71 (0.01 — 297.20)	0.537	0.84
APACHE II	0.051	-0.66	0.97 (0.87 — 1.07)	-0.034	0.51
Group (NMES vs. non-NMES)	0.759	-2.12	0.20 (0.05 — 0.89)	-1.609	0.03
Ventilator Weaning	0.813	-2.82	0.10 (0.02 — 0.50)	-2.289	0.01
Deep Sedation Period	0.055	-3.78	0.81 (0.73 — 0.90)	-0.208	< 0.01

Factors affecting physical function at discharge

Multiple linear regression analysis using the BI at discharge as the dependent variable revealed that the presence of ICU-AW was significantly associated with a lower BI (p < 0.01), whereas a higher IMS score and shorter time to active mobilization were significantly associated with a higher BI at discharge (p = 0.02 and 0.02, respectively; Table 4).

**Table 4:** Multiple linear regression results for factors affecting BI at discharge BI, barthel Index; ICU-AW, Intensive Care Unit-Acquired Weakness; IMS, Intensive Care Unit Mobility Scale; SMI, Skeletal Muscle mass Index; MRC, Medical Research Council score

Variable	Std. Error	t value	95% CI	Coefficient	p value
Constant	10.37	5.09	32.06 — 73.50	52.79	< 0.01
ICU-AW	7.26	-5.36	-53.41 — -24.40	-38.90	< 0.01
IMS	1.81	2.42	0.76 — 7.98	4.37	0.02
Time to active mobilization	0.24	-2.50	-1.07 — -0.12	-0.60	0.02

## Discussion

We investigated the impact of an early rehabilitation program using NMES during deep sedation on the prevention of ICU-AW and physical function at discharge in patients with ARDS. Our findings revealed that the incidence of ICU-AW was significantly lower in the NMES group. Furthermore, the implementation of NMES, ventilator weaning, and duration of deep sedation were predictors of ICU-AW, suggesting that the use of NMES is associated with a reduced risk of ICU-AW. Additionally, the presence of ICU-AW, IMS score, and time to active mobilization significantly affected the physical function at discharge. These results indicate that the prevention of ICU-AW and the promotion of early mobilization are crucial for improving functional independence at discharge.

Systematic reviews and meta-analyses on the effects of NMES on ICU-AW have not reached a consensus regarding its preventive effect on the onset of ICU-AW [7-9]. Moreover, the Rehabilitation Guidelines for Critically Ill Patients published in Japan also provide weak recommendations with low certainty of evidence [14]. However, limited investigations have focused on patients unable to exercise independently, specifically those under sedation. We hypothesized that combining NMES with early rehabilitation for ARDS, which requires careful intervention, would be beneficial for such patients. As hypothesized, our study demonstrated that NMES performed during deep sedation reduced the incidence of ICU-AW and increased physical activity levels within the ICU.

The pathophysiology of ICU-AW involves reduced excitability of muscle cell membranes owing to Na+ channel abnormalities [15], decreased Ca2+ ion release from the sarcoplasmic reticulum [16], and selective reduction of myosin [17,18]. These factors disrupt the muscle contraction mechanism, leading to muscle weakness. Additionally, in critically ill patients requiring deep sedation, both external stimuli (such as weight bearing and exercise) and internal stimuli (such as activation of contractile proteins) are eliminated, significantly affecting protein expression [19,20]. This mechanical silencing may influence the onset of ICU-AW. Previous reports have indicated that passive exercise using continuous passive motion devices in mechanically ventilated ICU patients reduced muscle atrophy [21] and partially mitigated muscle weakness [22,23]. However, these effects are limited, potentially owing to the lack of muscle action potentials, which are crucial for muscle contraction. NMES can induce electrical potential changes in muscles and provide mechanical stress. In our study, the NMES group showed greater preventive effects against ICU-AW compared to the non-NMES group, which received only passive exercise and stretching.

In patients who received NMES, it was feasible to perform the procedure even while they were under sedation and receiving muscle relaxants. Pain, a common issue associated with NMES, was effectively managed with sedatives and analgesics. In situations where muscle contraction was challenging owing to muscle relaxation, increasing the intensity of NMES successfully induced muscle contractions. Consequently, NMES may have helped prevent mechanical silencing, maintain the muscle contraction mechanism, and reduce the incidence of ICU-AW.

One study demonstrated that patients admitted to the emergency ward who received NMES experienced a 7% slower reduction in thigh muscle volume compared to those who did not receive NMES [24]. However, while NMES was effective in preventing muscle atrophy, it did not improve muscle strength or reduce the duration of mechanical ventilation [25]. In our study, there were no significant differences in skeletal muscle mass at ICU discharge between the two groups, and NMES did not contribute to a reduction in the duration of mechanical ventilation. These results partly differ from previous studies. In this study, NMES was performed under the administration of muscle relaxants in many cases, and although muscle contraction was observed, it was very weak. Therefore, it is possible that the contractions were insufficient to prevent muscle atrophy.

In our study, the implementation of NMES, ventilator weaning, and the duration of deep sedation were identified as significant predictors of the onset of ICU-AW. In patients requiring deep sedation, the duration of sedation cannot be shortened or avoided, and the ability to wean off the ventilator depends on respiratory function and lung recovery. These findings suggest that the early implementation of NMES as an early rehabilitation intervention is beneficial for ARDS patients with COVID-19 who require deep sedation.

We found that the presence of ICU-AW was associated with a significantly lower BI at discharge, indicating lower functional independence. Furthermore, a higher IMS score and a shorter time to active mobilization were significantly associated with a higher BI at discharge. However, some points need careful interpretation. Owing to the unique circumstances of the COVID-19 pandemic, the criteria for discharge and transfer differed from those during normal times (e.g., immediate transfer after extubation and sudden transfer decisions). As a result, the timing of physical function assessments at discharge varied, potentially affecting the results. Nevertheless, the study indicates that the presence of ICU-AW significantly affects the activities of daily living function at discharge, underscoring the importance of preventing ICU-AW. In early rehabilitation for ARDS, it is ideal to prevent the onset of ICU-AW and initiate exercise and mobilization as soon as the patient’s condition allows. These results support this ideal strategy and provide an important perspective in the early rehabilitation of patients with ARDS requiring long-term sedation.

In recent years, discussions on the long-term prognosis of critically ill patients admitted to the ICU and the improvement of post-intensive care syndrome have emphasized the importance of light sedation, appropriate nutrition, and early exercise [6,26]. Our study examined the effectiveness of NMES in critically ill patients with ARDS who required sedation, providing valuable insights for the early rehabilitation of future patients with ARDS.

The main limitations of this study include its retrospective design, small sample size, and single-center approach, which contribute to selection bias and limit the generalizability of the findings to other ICUs. Additionally, the study only evaluated the outcomes during the early hospital stay. Preventing physical function impairment is an emerging challenge in emergency and intensive care, with long-term quality of life and functional outcomes after discharge being increasingly emphasized [27]. As previously mentioned, the unique circumstances of the COVID-19 pandemic affected the criteria for discharge, transfer, hospital admission, and rehabilitation interventions, differing from normal times. It is necessary to consider the specific conditions and constraints of the pandemic when interpreting the study results [28-30].

## Conclusions

Early rehabilitation using NMES during deep sedation for critically ill patients with COVID-19 with ARDS may be associated with the onset of ICU-AW. In addition, the prevention of ICU-AW and the promotion of early mobilization were confirmed to contribute to improved functional independence at discharge. Although there are limited options for early rehabilitation in critically ill patients with ARDS for whom deep sedation cannot be avoided, NMES can be safely performed in sedated patients and may be beneficial in preventing ICU-AW. Further prospective study will be needed to elucidate the effect of early rehabilitation using NMES.
